# Neglected prognostic importance of ileal resection in patients with peritoneal metastasis

**DOI:** 10.1038/s41598-022-16100-x

**Published:** 2022-07-19

**Authors:** Tayfun Bisgin, Selman Sokmen, Berke Manoglu, Sevda Ozkardesler, Sulen Sarioglu, Hulya Ellidokuz

**Affiliations:** 1 Department of General Surgery, Dokuz Eylul University, Mithatpasa street no: 1606, Balcova, Izmir, Turkey; 2 Department of Anesthesiology and Reanimation, Dokuz Eylul University, Izmir, Turkey; 3 Department of Pathology, Dokuz Eylul University, Izmir, Turkey; 4 Department of Biostatistics, Dokuz Eylul University, Izmir, Turkey

**Keywords:** Gastrointestinal cancer, Gynaecological cancer, Mesothelioma, Metastasis

## Abstract

We aimed to determine the prognostic role of ileal resection on postoperative complications and the final oncological results of cytoreductive surgery (CRS) and hyperthermic intraperitoneal chemotherapy (HIPEC) treatment in patients with peritoneal metastasis (PM). Patients with PM who underwent CRS and HIPEC between 2007 and 2020 were analyzed retrospectively. Ileal resection was defined as the resection of the ileum at 100 cm or below. Patients were divided into ileal-resection and non-ileal resection groups. Besides clinico-pathological variables, peritoneal cancer index (PCI), completeness of cytoreduction (CC-0-1-2), (neo)adjuvant chemotherapy, operative time, need for surgical intensive care unit, and usage of blood products were all evaluated. The data of 664 patients was analyzed**.** Ileal resection was performed in 346(52.1%) patients. The median follow-up period was 27 months. The ileal resection group had significantly lower 3-and 5-year survival rates (55% and 43% vs. 69% and 52, *p* = .005, respectively). High PCI score (*p* < .001), more CC-1-2 cytoreductions (*p* < .001), more anastomoses (*p* < .001), prolonged operative time (*p* < .001), more ostomy creation (*p* = .001), increased morbidity (*p* < .001), and more infectious complications (*p* < .001) were all significantly associated with ileal resection. The loss of ileal function has a potential prognostic role in increased post-operative complications and worsened overall survival in patients with PMs.

## Introduction

Peritoneal metastasis (PM) is often considered as an end-stage disease. Cytoreductive surgery (CRS), hyperthermic intraperitoneal chemotherapy (HIPEC) and perioperative chemotherapy have emerged as the only potentially curative treatment of PM in the last three decades. In appropriately selected patients, this triplet treatment modality can be undertaken with low morbi-mortality and is associated with better survival outcomes^1–4^. Intraperitoneal disease burden is the most important prognostic factor^5^. It is well-known that this intraperitoneally disseminated disease burden prefers to settle down in particular areas such as the pouch of Douglas, sigmoid colon, terminal ileum, right paracolic gutter, and right subphrenic space, presumably by the combined effect of respiratory movements, gravity, limited mobility, and continuous bowel peristalsis^6–8^. Resection of the terminal ileum is often a component of aggressive CRS/HIPEC procedures. On the other hand, the ileum is the only segment of the small bowel in which bile acids and vitamin B12 are absorbed. It also has special capabilities for enzymatic digestion of nutrients, passive absorbtion of water, electrolytes, cholesterol, bicarbonate/chloride exchange, and immuno-protective functions^9–13^. Considering these unique functions of the ileum, the ileal resection may have a worsened effect on the postoperative outcomes. To the best of our knowledge, there is no solid data in the literature discussing the prognostic impact of loss of terminal ileum in this patient group with complex cancer care.

The objective of this study was to determine the prognostic role of ileal resection on postoperative complications and final oncological outcomes of CRS/HIPEC treatment in PM.

## Material and methods

A prospectively recorded database of consecutive CRS and HIPEC procedures performed in the Peritoneal Surface Malignancy Center between 2007 and 2020 was reviewed. Patients who had undergone ileal resection (< 100 cm) were included. The exclusion criteria for CRS and HIPEC were: (1) massive involvement of the retroperitoneum, (2) mesenteric pedicle invaion, (3) massive small bowel involvement (which would result in a short bowel after resection), (4) unresectable extra-abdominal metastasis, (5) major vascular invasion, and (6) low ECOG perfomance status. All procedures performed in this study were in accordance with the ethical standards of the institutional and/or national research committee and with the 1964 Helsinki declaration and its later amendments or comparable ethical standards. The study was approved by the local ethics committee. Informed consents was received from all of the patients.

The demographic data, tumor characteristics, extent of PM, intraoperative findings, and postoperative results were gathered from the medical records. Patients were divided into ileal-resection and non-ileal resection groups, and postoperative overall morbidity, mortality, and oncologic outcomes were analyzed.

### Preoperative assessment

All patients who were potentially candidates for CRS/HIPEC were discussed on the multidisciplinary tumor board. Thoraco-abdominal computed tomography (CT), and magnetic resonance imaging (MRI), and/or positron emission tomography-CT, if needed, were used for preoperative radiologic evaluation. The co-morbidities were recorded according to the Charlson co-morbidity index (CCI)^14^. The interval between the last chemotherapy dose and surgery was at least 4 weeks in patients who received neoadjuvant treatment.

### Cytoreductive surgery and hyperthermic intraperitoneal chemotherapy

Mechanical bowel preparation and venous thromboembolism prophylaxis were given to all of the patients. Intravenous antibiotics (cefuroxime axetil and metronidazole) were administered 30 min before the incision and repeated every 3 h. Cytoreductive surgery was performed by the same surgical team as described by Sugarbaker, allowing the eradication of all macroscopic tumors^15^. The extent of the disease was assessed by the peritoneal carcinomatosis index (PCI)^16^. In the intraoperative evaluation, ileal resection was performed if the ileum was involved with disease that would not allow complete cytoreduction. Residual disease was recorded according to the ''Completeness of cytoreduction'' score: no residual tumor, CC-0; residual tumor ≤ 2.5 mm, CC-1; residual tumor > 2.5 mm, CC-2^17^.

After CRS, two inflow (one in the pevis, one in the subhepatic area) and two outflow drains and two thermal probes were positioned in the abdominal cavity, and HIPEC was delivered by a perfusion system (The Belmont® Rapid Infuser RI-2, MA, USA) with the closed abdominal technique. The chemotherapeutic agents were decided by our oncologist individually for every patient. At a constant temperature of 42.5 °C, oxaliplatin (400 mg/m^2^, 30 min), mitomycin-C (10 mg/m^2^) and/or cisplatin (75 mg/m^2^, 90 min) were administered. Patients did not receive concurrent intravenous chemotherapy. Gastrointestinal or urinary anastomoses were performed before HIPEC.

### Early postoperative care and follow-up

"Common Terminology Criteria for Adverse Events" was used to record postoperative morbidity and HIPEC toxicity (minor morbidity-grade I/II, major morbidity-grade III/IV, or mortality-grade V^18^. Postoperative complications were classified according to the Clavien-Dindo system^19^. Death within 30 days after surgery and in-hospital mortality were recorded as mortality.

For the first year, physical examination and CEA measurements were performed every three months, and thoraco-abdominal computed tomography every six months. For the second year, physical examination and CEA measurements were performed twice a year and computed tomography once a year. Patients underwent colonoscopy at the end of the first year.

### Statistical analysis

Statistical analysis was performed using SPSS 22.0. Categorical variables were compared among groups using the Pearson Chi-Square test. Continuous variables were compared by independent samples *t*-test. Continuous variables were expressed as means and ranges, and categorical variables as frequencies and percentages. Univariate analysis was performed using the Chi-Square test, and multivariate analysis was performed using a binary logistic regression model. Survival rates were calculated using the Kaplan–Meier method and were compared with the log-rank test. Multivariate analysis to identify predictors of survival was performed by constructing stepwise Cox proportional hazard models incorporating variables selected on the basis of the results of univariate analysis. P values < 0.05 were defined as statistically significant.

## Results

### Patients characteristics

There were 664 consecutive CRS/HIPEC patients between 2007 and 2020, among whom 346 (52.1%) were in the ileal resection group and 318 (47.9%) were in the non-ileal resection group. The mean age was 54.5 (± 17.3) years. Median follow-up and survival were 27 (1–190) and 55 (44–67) months, respectively. The origin of PM was ovarian in 280 (40.3%), colorectal in 212 (30.9%), appendix in 74 (11.2%), peritoneal mesothelioma in 37 (5.6%), stomach in 26 (3.9%), and other tumors in 35 (5.3%). Ovarian cancer-PM was similarly distributed between groups; colorectal cancer and appendicieal cancer-PM were more common in the ileal-resection group (*p* = 0.005). The clinico-demographic findings of patients are given in Table 1.

**Table 1:** Clinical and demographic characteristics of the patients.

	Ileal resection, n (%) 346 (52.1)	Non-ileal resection, n (%) 318 (47.9)	p*
**Gender**			**< .001**
Male	110 (16.6)	63 (9.5)	
Female	236 (35.5)	255 (38.4)	
Age (year, mean ± SD)	55.3 ± 20.8	53.7 ± 12.3	*.218*
BMI (mean ± SD)	27.7 ± 11.1	29.9 ± 20.3	*.279*
Smoking (+)	74 (11.2)	59 (9.0)	*.324*
Co-morbity	150 (22.6)	134 (20.2)	*.752*
**Tumoral origin**			**.005**
Ovarian	128 (19.3)	152 (22.9)	
Colorectal	123 (18.5)	89(12.4)	
Appendiceal	51 (7.7)	23 (3.5)	
Peritoneal mesothelioma	18 (2.7)	19 (2.9)	
Gastric	10 (1.5)	16 (2.4)	
Others	16 (2.4)	19 (2.9)	
**Onset**			**.001**
Synchronous	185 (29.6)	131 (20.9)	
Metachronous	141 (22.5)	169 (27.0)	
Neoadjuvant chemotherapy (+)	200 (30.1)	219 (33.0)	**.003**

Bold values indicated statistical significance (*p* < 0.005).

*BMI* body mass index.

*Pearson χ^2^ test and independent samples *t*-test.

Significant values are in italics.

Univariate analysis showed that high PCI (PCI ≥ 15) (*p* < 0.001), CC 1–2 cytoreduction (*p* < 0.001), 5 or more organ resections (*p* < 0.001), 1 or more anastomoses (*p* < 0.001), prolonged operative time (*p* < 0.001), and ostomy creation (*p* = 0.001) were significantly associated with ileal resection. The ileal resection group had higher rates of both grade I–II and grade III–IV morbidity (*p* < 0.001) and perioperative mortality (*p* < 0.001). Ileal resection was associated with more infectious complications (*p* < 0.001) (Table 2). Male gender (OR, 1.81; 95% CI 1.22–2.68; *p* = 0.003), high PCI score (OR, 4.10; 95% CI 2.86–5.89; *p* < 0.001), and neoadjuvant chemotherapy (OR, 1.51; 95% CI 1.07–2.14; *p* = 0.020) were found to be significant risk factors in the ileal resection group in multivariate analysis (Table 3).

**Table 2:** Comparison of surgical characteristics between ileal resection and non-ileal resection groups.

	Ileal resection, n (%) 346 (52.1)	Non-ileal resection, n (%) 318 (47.9)	p*
**Perioperative characteristics**			
**PCI**			**< .001**
< 15	177 (26.7)	258 (38.9)	
≥ 15	169 (25.5)	60 (9.0)	
**Completeness of cytoreduction**			**< .001**
CC-0	212 (33.9)	252 (40.3)	
CC-1,2	114 (18.2)	48 (7.7)	
**Number of resected organs**			**< .001**
< 5	179 (27.0)	300 (45.2)	
≥ 5	167 (25.2)	18 (2.7)	
**Number of resected anatomosis**			**< .001**
0	111 (16.5)	178 (26.8)	
1	184 (22.7)	116 (17.5)	
≥ 2	51 (7.7)	24 (3.4)	
Ostomy (+)	154 (23.5)	50 (7.6)	**< .001**
Preoperative RBCs (+)	22 (3.3)	10 (1.5)	*.052*
Intraoperative RBCs (+)	175 (26.6)	99 (15.0)	**< .001**
Intraoperative albumin (+)	83 (12.7)	36 (5.5)	**< .001**
Intraoperative FFP (+)	139 (22.2)	69 (11.6)	**< .001**
Operative time (min, mean ± SD)	358.6 ± 114.1	320 ± 12.3	**< .001**
**Postoperative characteristics**			
ICU stay (+)	123 (18.9)	103 (15.5)	*.391*
Post-operative RBCs (+)	82 (12.6)	36 (5.5)	**< .001**
Length of hospital stay (days, mean ± SD)	21.2 ± 14.5	17.7 ± 13.8	**.004**
**Morbidity**			**< .001**
None	204 (30.7)	238 (35.8)	
Grade I–II	66 (9.9)	38 (5.7)	
Grade III–IV	52 (7.8)	37 (5.6)	
Postoperative mortality	24 (3.6)	5 (0.8)	**< .001**
HIPEC toxicity	38 (5.7)	25 (3.8)	*.167*
Infection (+)	100 (15.2)	43 (6.5)	**< .001**
Nephrotoxicity (+)	40 (11.6)	21 (6.6)	**.027**

Bold values indicated statistical significance (*p* < 0.005).

*PCI* peritoneal carcinomatosis index, *RBC* red blood cell, *FFP* fresh frozen plasma, *ICU* intensive care unit, *HIPEC* Hyperthermic intraperitoneal chemotherapy.

*Pearson χ^2^ test and independent samples *t* test.

Significant values are in italics.

**Table 3:** Preoperative factors independently associated with ileal resection in multivariate analysis.

	OR	95% CI	*p*
Gender (male)	1.81	1.22–2.68	**.003**
**PCI**			
≥ 15	4.10	2.86–5.89	< *.001*
Neoadjuvant chemotherapy (+)	1.51	1.07–2.14	**.020**

*PCI* peritoneal carcinomatosis index.

Significant values are in bold and italics.

The ileal resection group had significantly lower 3- and 5-year overall survival rates (55% and 69% vs. 43% and 52, *p* = 0.005, respectively) (Table 4). Significant factors such as ileal resection, PCI score, complete cytoreduction, the use of intraoperative blood products, HIPEC toxicity, and infection were modeled into Cox regression analysis to determine independent prognostic factors of survival. High PCI score (HR, 1.41; 95% CI 1.11–1.80; *p* = 0.002), complete cytoreduction (CC-0vsCC-1-2) (HR, 1.52; 95% CI 1.19–1.95; *p* = 0.001), intraoperative ES (HR, 1.63; 95% CI 1.30–2.07; *p* < 0.001), and infection (HR,1.52; 95% CI 1.19–1.95; *p* = 0.001) were independent prognostic factors in Cox-regression analysis (Table 5).

**Table 4:**
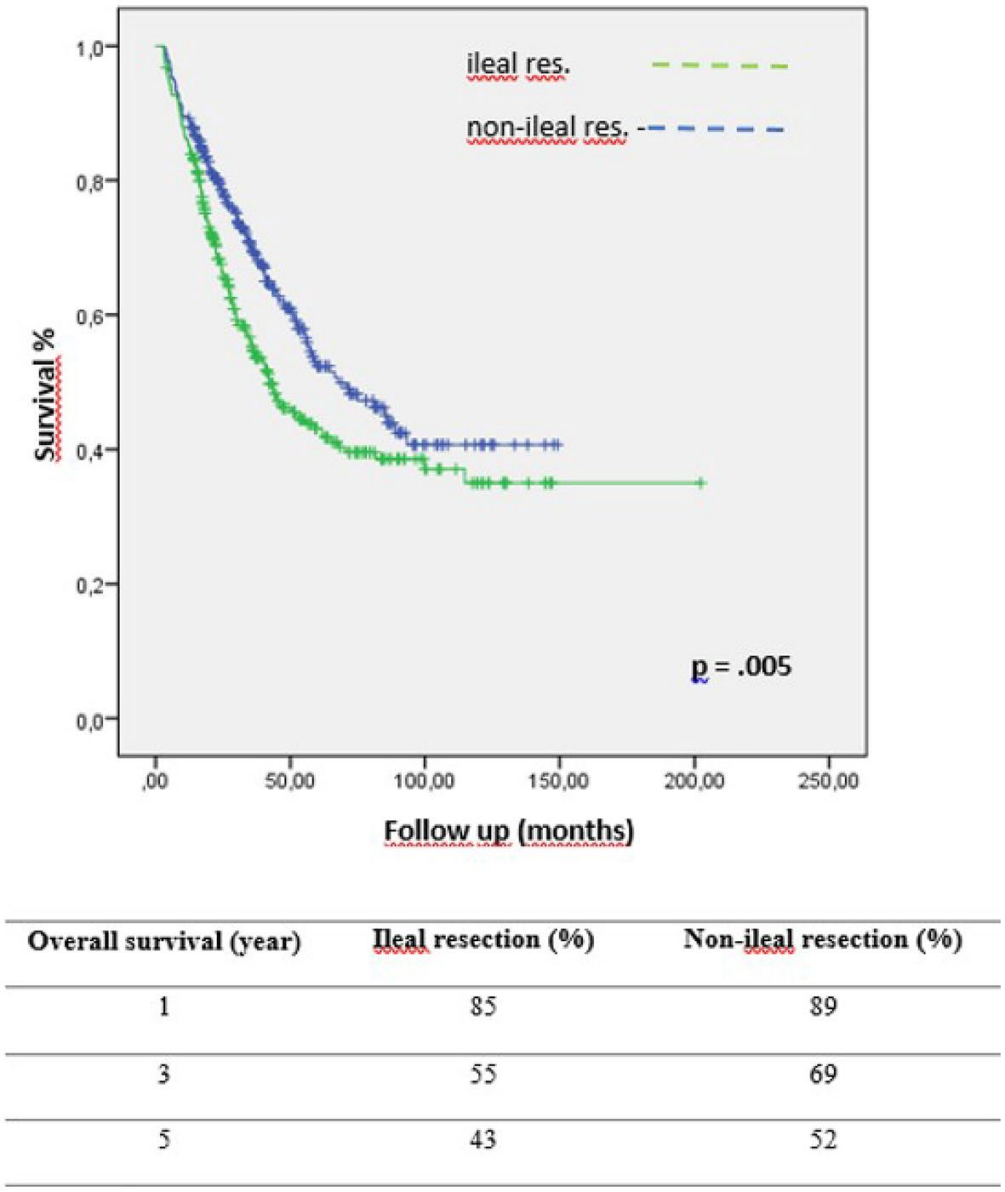
Overall survival of the patients between two groups.

**Table 5:** Independent prognostic factors in Cox-regression analysis.

	HR	95% CI	*p*
PCI (≥ 15)	1.41	1.11–1.80	**.002**
Completeness of cytoreduction	1.52	1.19–1.95	**.001**
Intraoperative ES	1.63	1.30–2.07	**< .001**
Infection	1.52	1.19–1.95	**.001**

Significant values are in bold.

## Discussion

For the last three decades, the prognosis of patients with PM has been improved by multimodal treatment, including complete cytoreduction and HIPEC. This combined therapy has prolonged overall survival rates in carefully selected patients. The prognosis of these patients primarily depends on the extent of the peritoneal disease (PCI), the completeness of cytoreduction, and the histopathologic types^1–5^. To achieve complete resection, an extended multivisceral and/or peritoneal resection is usually needed. On the other hand, the degree of small bowel involvement is insistently considered to be a limiting factor to radically eradicate macroscopic tumor burden^8,20–22^. The terminal ileum, as a special part of the small bowel with its own features of anatomical fixity and limited peristaltic movements, is frequently resected in cytoreductive procedures. Besides, the intraperitoneal fluid circulates in a well-defined manner (in the head-caudal-head direction and is controlled by gravity and respiratory movement sequence), which leads to the accumulation of influxing cells on some special anatomic sites such as the ileum^6–8^. In light of these findings, the terminal ileum can be affected by tumor nodules irrespective of the extent of the disease. The current study is particularly focused on the outcomes of ileal resection in patients with PM. High PCI, more CC-1 or CC-2 resection, more gastrointestinal anastomosis, more ostomy formation, more blood products, and albumin usage were strikingly in the ileal resection group. In addition, ileum-resected patients had prolonged operative time, high post-operative infection, increased nephrotoxicity, and prolonged hospital stay. C–D grade I–IV morbidity and perioperative mortality were significantly higher in the resection group.

The clinical consequences of functional loss of the terminal ileum and ileocecal valve have been well-documented in the literature: the ileocecal valve acts as a structural barrier for the passage of intestinal contents and provides the required time for efficient absorption of nutrients, electrolytes, and water by slowing intestinal transit time^13^. Bile acids, vitamin B12, and fat-soluble vitamins (vitamins A, D, E, and K) are significantly absorbed in the last 100 cm of ileum^9–13^. These vitamins are a prerequisite for normal cell division and growth. The loss of these important mechanisms may possibly result in a decline in the physiological reserve and immuncompetence of the patient and may contribute to more infections, more grade I–II and grade III–IV morbidity, and longer hospital stays in the ileal resection group in our series. The ileum is also the main site of bicarbonate and chloride exchange and has an important clinical role in the passive absorption of water and electrolytes. Only the ileum can absorb sodium chloride against steep electrochemical gradients. When the ileal mechanism that provides the exchange of bicarbonate with chloride ions is disrupted, excessive amounts of chloride and hydrogen ions pass into the colon, bicarbonate accumulates in the body and metabolic alkalosis develops. When the decrease in bicarbonate/chloride exchange is accompanied by loss of fluid (diarrhea) and monovalent ions, the kidneys cannot maintain the acid–base balance and alkalosis deepens^13,23^. Considering these unique tasks of the ileum, nephrotoxicity was found to be a statistically significant factor in the ileal resection group in our series. It’s sure that chemotherapeutic agents used during HIPEC also had an important role in nephrotoxicity, but 40 of the 61 (67%) patients who developed nephrotoxicity were patients with ileal resection (*p* = 0.027). In this nephrotoxicity group, cisplatin was used in 19 of these patients, cisplatin + mitomycin in 27, oxaloplatin in 8, and other chemotherapeutic agents in 5 patients.

Hence, resection of the ileum may adversely affect overall survival. In our study, the ileal resection group had significantly lower 1-, 3-, and 5-year survival rates (*p* = 0.005). Distal small bowel resections are less tolerated than proximal small bowel resections. This important feature is because of the remarkable ability of the ileum to compansate the absorbtive functions of the proximal intestine. Fifty to sixty percent of the mid-jejunum can be resected with few long-term metabolic consequences, however, resection of more than 30% of the ileum is poorly tolerated^24–26^. It is well known that tumor burden (PCI) and complete cytoreduction are the most important prognostic factors in peritoneal metastasis. In our study, high PCI, complete cytoreduction, intraoperative ES replacement, and post-operative infection were independent prognostic factors for overall survival in Cox regression analysis whereas ileal resection could not stay in the Cox model. Higher PCI scores and incomplete cytoreduction rates in the ileal resection group may undoubtedly lead to lower survival rates. We are fully aware that it is pleonastic to underline that the assessment of terminal ileum involvement partly contributes to overall PCI assessment. However, in many studies, heterogeneous PCI cut-off scores have been accepted as a prognostic role in peritoneal metastasis. In addition, the involvement of some special areas (such as small bowel) negatively affects both the probability of a complete cytoreduction and the prognosis, regardless of the PCI score. Elias et al., clearly demonstrated that, independent of PCI, terminal ileal segment involvement in the small intestine has a negative effect on the prognosis. They revealed that lower ileal segment involvement, whether PCI < 15 or PCI ≥ 15, significantly worsened 5-year overall survival for peritoneal metastasis of colorectal origin^8^. Also, we are fully aware that the inclusion of tumors from different origins is one of the limitations of the study and they each have different survival rates. However, we aimed to show the effects of ileal resection on post-operative complications in a large case series. In the light of all this, it would be assertive to say that ileal resection alone is associated with poor survival. But the above-mentioned unique functions of the ileum, which are not replaceable in the long-term period, may be one of the key factors that negatively affect post-operative morbi-mortality and overall survival in ileal resection group. In a global assessment, the involvement of terminal ileum should be added amongst the considered factors to precisely evaluate every patient's prognosis.

To the best of our knowledge, this is the first study reporting outcomes of ileal resection in patients with PM who underwent CRS and HIPEC. It has several inherent limitations. First, it was an observational, retropro mono-center study. Second, there were various histotypes of peritoneal surface malignancies. And also, potential confounding factors from uncontrollable variables may affected the analyses. We are fully aware that ileum-resected patients usually undergo more extensive cytoreductive procedures. Thus, it can be pleonastic to overstress the assessment of the ileum as a risk factor besides the well-known variables. But, we aimed to search for the clinical significance of ileal resection in high volume patient data to improve results to optimize patients’ intensive care and oncologic outcomes.

## Conclusion

In this study, the ileum was resected in 52% of cytoreduced patients. Ileal resection negatively affected morbi-mortality results and oncologic outcomes. These consequences of ileal resection are neglected in both clinical practice and the related literature. To optimize outcomes, prehabilitation, vigilant monitoring, intense care, and increased awareness must be instituted for these complex cancer patients.

## Data Availability

The datasets used and/or analysed during the current study available from the corresponding author on reasonable request.
